# King Lear Fighting the Dementors: Rethinking the Politics of Dementia Representation in Popular Culture

**DOI:** 10.1093/geroni/igaf122.686

**Published:** 2025-12-31

**Authors:** Ulla Kriebernegg, Heike Hartung

**Affiliations:** University of Graz, Graz, Steiermark, Austria; University of Potsdam, Potsdam, Brandenburg, Germany

## Abstract

This paper examines how contemporary literature, film, and theater shape public perceptions of dementia, often reinforcing negative stereotypes that contribute to the othering of individuals living with the condition. Popular fiction, such as J.K. Rowling’s Harry Potter series, employs dementia-adjacent metaphors—like the soul-sucking Dementors—to depict memory loss as an erasure of identity. Similarly, films like Still Alice and The Father use dementia metonymically, reducing individuals to symbols of loss. Since the 1980s, cultural representations of dementia have ranged from depictions of the disease as a “living death” to more nuanced explorations of forgetfulness. However, today, dementia is frequently framed as a crisis of aging, often through symbolic narratives that heighten societal anxieties about cognitive decline. A key example that will be analyzed is Shakespeare’s King Lear, whose modern reinterpretations increasingly medicalize Lear’s descent into madness, diagnosing him with Lewy Body Dementia. This reading shifts the focus from Lear’s emotional and psychological turmoil to an ageist burden narrative that aligns with contemporary fears of cognitive decline, loss of autonomy, and aging as a social and economic crisis. This paper first analyzes these culturally influential examples to explore how mainstream narratives shape public discourse on dementia. It then turns to more nuanced portrayals to examine aesthetic strategies that offer alternative and more humane representations. Ultimately, we argue for a cultural politics of dementia informed by critical aging studies to challenge ageist frameworks and promote attitudinal change toward aging and dementia.

